# mTOR as a Potential Target for the Treatment of Microbial Infections, Inflammatory Bowel Diseases, and Colorectal Cancer

**DOI:** 10.3390/ijms232012470

**Published:** 2022-10-18

**Authors:** Obaid Afzal, Abdulmalik S. A. Altamimi, Bismillah Mubeen, Sami I. Alzarea, Waleed Hassan Almalki, Salwa D. Al-Qahtani, Eman M. Atiya, Fahad A. Al-Abbasi, Fatima Ali, Inam Ullah, Muhammad Shahid Nadeem, Imran Kazmi

**Affiliations:** 1 Department of Pharmaceutical Chemistry, College of Pharmacy, Prince Sattam Bin Abdulaziz University, Al-Kharj 11942, Saudi Arabia; 2 Institute of Molecular Biology and Biotechnology, The University of Lahore, Lahore 54590, Pakistan; 3 Department of Pharmacology, College of Pharmacy, Jouf University, Sakaka 72341, Saudi Arabia; 4 Department of Pharmacology, College of Pharmacy, Umm Al-Qura University, Makkah 21955, Saudi Arabia; 5 Department of Medical Laboratory Sciences, Faculty of Applied Medical Science, Majmaah University, Al-Majmaah 11952, Saudi Arabia; 6 Department of Biochemistry, Faculty of Science, King Abdulaziz University, Jeddah 21589, Saudi Arabia

**Keywords:** mammalian target of rapamycin (mTOR), inflammatory bowel diseases (IBD), colorectal cancer (CRC)

## Abstract

The mammalian target of rapamycin (mTOR) is the major controller of a number of important cellular activities, including protein synthesis, cell expansion, multiplication, autophagy, lysosomal function, and cellular metabolism. When mTOR interacts with specific adaptor proteins, it forms two complexes, mTOR complex 1 (mTORC1) and mTOR complex 2 (mTORC2). The mTOR signaling system regulates gene transcription and protein manufacturing to control proliferation of cell, differentiation of immune cell, and tumor metabolism. Due to its vital role in case of microbial infections, inflammations and cancer development and progression, mTOR has been considered as a key therapeutic target for the development of targeted medication. As autophagy dysfunction is linked to changes in both innate and adaptive immune responses, bacterial clearance defects, and goblet and Paneth cell malfunction, all of these changes are linked to inflammatory bowel diseases (IBD) and colorectal cancer (CRC) pathogenesis. Preclinical and clinical data have shown that the inhibition and induction of autophagy have significant potential to be translated into the clinical applications. In IBD and several CRC models, mTORC1 inhibitors have been found effective. In the recent years, a number of novel mTOR inhibitors have been investigated in clinical trials, and a number of drugs have shown considerably enhanced efficacy when combined with mTOR inhibitors. The future developments in the mTOR targeting medications can benefit patients in individualized therapy. Advanced and innovative medicines that are more effective and have lower drug resistance are still in high demand. New findings could be relevant in medicine development, pharmacological modification, or future mTOR inhibitor research. Therefore, the goal of this review is to present a comprehensive account of current developments on the mTOR pathway and its inhibitors, with an emphasis on the management of microbial infections, the treatment of inflammatory bowel disease, and the management of colon cancer.

## 1. Introduction

The mammalian target of rapamycin family of serine-threonine protein kinases is abbreviated as mTOR. The fundamental function in the cell of these kinases is to regulate the level of catabolic and anabolic processes by combining external information with evidence about metabolic resources of cell. mTOR is present in two heteromeric protein complexes in mammalian cells which are called mTOR complex 1 (mTORC1) and mTOR complex 2 (mTORC2) [1,2]. The biological process driving mTOR activity is the phosphorylation of two key regulators of protein translation, p70 ribosomal S6 protein kinase (p70S6K) and eIF-4E binding protein (4E-BP) [3,4]. mTORC1 regulates transcription and translation, cell cycle, autophagy, and microtubule dynamics whereas the mTORC2 regulates the activity of two protein kinases, Akt and PKCa [5,6]. It controls the activity of Akt by phosphorylating serine 473 [7]. In the canonical route, mitogens, trophic chemicals, and hormones all activate mTOR. Phosphoinositide-30 kinase (PI3K) is activated and phosphatidylinositol 3,4,5-triphosphate (PIP3) production is improved as a result of the involvement of SH2 domain containing adaptor proteins and Ras (rat sarcoma) which is a small G protein [8,9]. Improved PIP3 levels cause the conscription of 3-phosphoinosititide-dependent protein kinase 1 (PDK1) and Akt on the membrane of the cell, as well as consequent phosphorylation of Akt by PDK1 and mTORC2 [10,11]. In addition to its critical roles in the complex signaling pathways, mTOR is also required for a variety of physiological functions in the human adult brain, including synaptic plasticity, learning, memory, and food absorption control. The pathway is important in neuronal growth, dendritic development, and spine morphogenesis, among other things [12]. Several factors such as mitogens, amino acids, trophic factors and hormones as well as pathological situations viz heat shock, ischemia, DNA damage, cellular stress, and viral infections, all influence the kinase activity of downstream components of the mTOR pathway [13,14]. As a result, the activity of the mTOR kinase regulates a variety of cellular processes that can have both good and negative consequences, for instance enhanced protein translation and decreased cell viability, by activating autophagy processes [15].

In reaction to a variety of intracellular and extracellular signals, together with glucose, cytokines, amino acids, and PAMPs (pathogen-associated molecular patterns), mTOR displays critical role in development, cellular proliferation, immunology, and cancer [16,17]. This pathway can contribute to autophagy suppression by phosphorylating and inactivating the autophagy initiator ULK1 in nutrient-sufficient circumstances [18]. New data are linking mTOR to intestinal homeostasis and cancer [19]. The genetic ablation of mTOR in intestinal epithelial cells (IEC) demonstrated that mTOR plays a vital part in the regulation of caloric restriction, Paneth/goblet cell formation, and altered epithelial proliferation [17]. It presents the link between mTOR dysregulation, chronic inflammation, and colon cancer. According to Gutierrez-Martnez et al., 2019, mTORC1 signaling prevents epithelial DNA damage, downstream of interferon (IFN), keeping intestinal epithelial cells stable and preventing cancer development in colitis, an inflammatory bowel disease (IBD) that reasons ulcers and inflammation in the digestive tract [18]. When supplies of energy are scarce, mTOR action is suppressed, biosynthesis is slowed, and autophagy is activated to reprocess the resources rather than producing new ones [19], translational halt and cell death are avoided as a result. When nutrients and energy are abundant, mTOR is activated, signaling downstream pathways to produce new cellular material, promoting cellular growth and proliferation though reducing autophagy. As autophagy speeds up the recycling of cellular components that are no longer needed or are damaged. These cellular constituents are encased in a double-membraned vesicle, which is subsequently directed to a lysosome, destroyed, and recycled [20]. As intracellular infections compete with the host for energy and resources, autophagy control by mTOR serves as a bridge between pathogen attack and host defense [21]. By regulating activation, development and effector functions of lymphoid cells, mTOR helps to facilitate immunological protection against invading pathogens [22]. The AMPK (AMP-activated protein kinase)/mTOR (adenosine monophosphate activated protein kinase/mTOR) signaling pathway, the PI3K/Akt/mTOR signaling pathway and other autophagy signaling pathway are primarily responsible for autophagy. Autophagy dysfunction can have health and cancer ramifications also. Autophagy has a dual effect on cancer, limiting tumor genesis and also promoting tumor growth [23]. In eukaryotes, cellular homeostasis is maintained by ubiquitin-protease system (UPS) and autophagy. Although both systems use ubiquitination as a deterioration signal, the mechanisms involved are distinct [24]. The UPS degrades short-term proteins and misfolded proteins, while autophagy removes abiding proteins, insoluble protein masses, and whole organelles (e.g., mitochondria, peroxisomes) as well as intracellular parasites (e.g., bacteria) [25,26]. Recent studies have revealed the existence of links and mutual control mechanisms between these degradation pathways through mTOR activation and inactivation, in addition to an indirect connection through ubiquitylated proteins [27,28]. In addition, mTOR signaling system is convoluted in the majority of human malignancies [29,30,31]. The pathway, has also been linked to intestinal homeostasis, according to new findings [32,33,34].

Beneficial effects of TORC1 inhibitors have been reported in IBD and various CRC models [35]. Colorectal cancer (CRC) is the world’s third biggest cause of cancer-related mortality. In spite of the rising armament of targeted and chemotherapeutic drugs, the complete five years of survival frequency for cancer patients at the metastatic stage is less than ten [36]. Prognosis and treatment of advanced CRC remain difficult clinical research priorities [37]. mTOR is a serine/threonine protein kinase that controls cellular growth and proliferation by stimulating critical anabolic processes, detecting nutrients and hormones for growth, and responding to diverse environmental signals [38]. It has been proved through in vitro and preclinical investigations that mTOR is an effective target for colorectal cancer therapy [34,39]. It has been discovered that mTOR signaling plays a significant role in colorectal cancer. In cancer models of mice, inhibiting mTOR substantially decreased intestinal carcinogenesis and increased survival [34]. The AMPK/mTOR signaling system also has provided numerous prospective therapeutic targets for colorectal cancer [40]. To date, first and second generations of mTOR inhibitors have been introduced for the treatment of cancer. Antitumor effects of mTOR inhibitors are linked to tumor development, angiogenesis, and invasion inhibition. In vivo, mTOR inhibitors, notably rapalogs, cause cancer cell death, but not at dosages that decrease cell proliferation in culture [29]. Rapamycin, a selective inhibitor of the (mTOR), is a promising cancer therapeutic option [30,31]. In this context, we aimed to provide an accumulative account of recent updates on mTOR pathway and its inhibitors with special focus on the microbial infection control, inflammatory bowel disease treatment, and colon cancer treatment.

## 2. mTOR Pathway at a Glance

mTOR pathway is an important regulator and controller of physiology and mammalian metabolism, with major functions in the functioning of organs such as muscles, liver, white/brown adipose tissue, and brain. It is disrupted in disorders such as obesity, diabetes, depression, and malignancies [41]. mTOR protein kinase is crucial to understanding one of biology’s most fundamental questions: What mechanisms do organisms use to control their growth and coordinate it with nutrition availability. It mediates most anabolic and catabolic processes in response to nutrition and nutrient-induced signals, as the catalytic subunit of two different protein complexes, mTORC1 and mTORC2 [42]. mTORC1 is made up of mTOR, regulatory-associated protein of mammalian target of rapamycin (RAPTOR), prolinerich Akt substrate 40 (PRAS40), and MTOR-associated protein, LST8 Homolog (mLST8). mTORC2 is made up of mTOR, mLST8, RICTOR (rapamycin-insensitive companion of mammalian target of rapamycin), mSIN1, and Protor-1. It regulates cell proliferation and endurance by phosphorylating and activating the Akt kinase [43].

**Figure 1:**
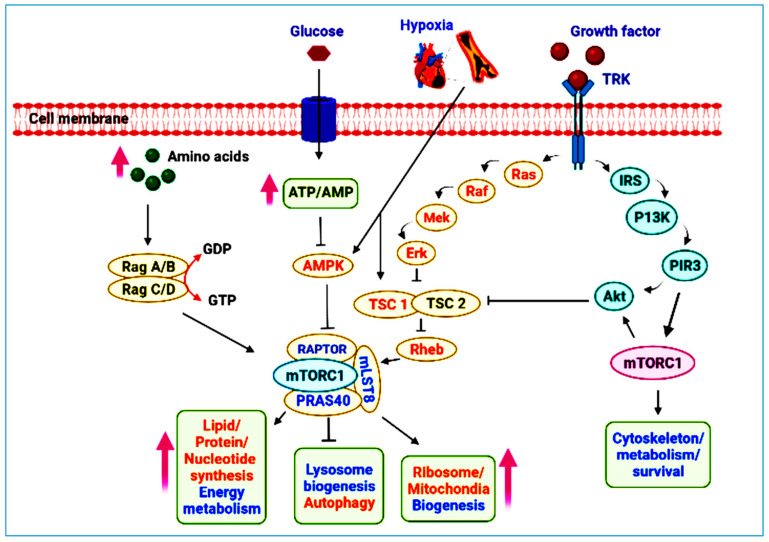
The structural components, types, and physiological role of mTOR signaling pathway. Upward and downward direction of arrows indicate the increase or decrease of process or substance. When growth factor stimulates TRK receptor then it triggers the downregulation of Ras, Raf Mek, Erk, IRS, P13k, PIR3, and Akt which ultimately cause inhibition of TSC1/TSC2 complex which ultimately inhibits lysosome biogenesis autophagy and upregulates the process of ribosome/mitochondria biogenesis and lipid/protein/nucleotide synthesis and energy metabolism by inhibiting Rheb. On the other hand, Rag A/B and Rag C/D cause the same effect by amino acids upregulation.

The mTOR genes were first of all discovered in yeast, later its role in cell proliferation and synthesis of protein was described. Peterson and Sabatini groups created the first mTOR-deleted mouse model, demonstrating that mTOR is critical for survival and development. Subsequently, the mTOR signaling pathway was studied using both genetic and biochemical methods [44]. The discovery that FK506 and rapamycin decrease separate elements of immune cell activity and antagonize each other when administered jointly offered early insights into rapamycin’s mode of action, proposing that, regardless of their structural similarities, they target different objectives [45,46]. When Stuart L. Schreiber discovered that FK506 and rapamycin impasse to the equivalent location on FKBP12 (FK506-binding proteins) [47], the founder member of the FKBP family of immunophillins, he made a huge breakthrough (proteins that bind immunosuppressants). He postulated that FKBP12-FK506 and FKBP12-rapamycin both act through a gain-of-function mechanism in which they bind to different protein targets. Soon after, he discovered that FKBP12—FK506 suppresses the phosphatase calcineurin, which dephosphorylates and thereby initiates transcription factor NEATc, and that this explains FK506′s cell biology and signaling actions [48]. Because sequence research revealed that RAFT1/FRAP/mTOR shared similarity, chiefly kinase domain of it, with the protein encoded by the TOR1/DRR1 and TOR2/DRR2 genes of budding yeast, Abraham devised the term mTOR [42]. mTOR is a member of the phosphatidylinositol—kinase related kinase family (PI3K). This family’s members are a big (>2500 amino acids) and have kinase domain at their C-termini that is comparable in order to phosphatidylinositol-3-kinase (PI3K) [47]. TORC1 a key constituent of the PI3K/Akt pathway, relaying indications from tumor suppressors phosphatase and Tensin Homolog deleted on Chromosome 10 (PTEN), tuberous sclerosis complex TSC1/2 (tuberous sclerosis complex 1/2) and LKB1 as well as carcinogenic proteins PI3K and Akt [49]. mTORC1 regulates protein synthesis and turnover downstream, which governs cellular biogenesis. It phosphorylates two components involved in translation initiation: eIF4E binding protein 1 (4EBP1) and ribosomal protein S6 kinase (S6K) [50]. By inhibiting autophagy, it regulates protein turnover [51]. mTORC2, like mTORC1, is engaged in the PI3K/Akt pathway; however, it has a different role than mTORC1. It phosphorylates and increases AKT activation, making it important for cell survival mediated by Akt [52]. The activation of PI3K is triggered by the ligand bound eliciting one of the transmembrane receptors. Following that, PI3K phosphorylates Akt, which PTEN then dephosphorylates. PTEN deficiency is linked to a worse prognosis in NSCLC, which is most likely due to increased PI3K/Akt/mTOR pathway downstream signaling [53]. mTORC1 and mTORC2 are two mTOR complexes that regulate cell proliferation [54]. Rapamycin partially inhibits mTORC1, it unifies multiple signals that indicate the availability of growth factors, nutrients, and energy in order to promote cellular growth and catabolic processes during stress [55,56]. Akt is used by growth factors and hormones to signal mTORC1, which deactivates tuberous sclerosis complex 2 to prevent mTORC1 inhibition [57]. Activated mTORC1 has a variety of biological consequences, comprising mRNA translation via phosphorylation of downstream targets such as 4E-BPI and p70 S6 kinase, autophagy overpowering via Atg13 and ULK1, ribosome biogenesis and transcription stimulation that primes to improved activity of mitochondria [58]. mTORC2 increases cell survival by activating Akt [59]. By activating PKC and phosphorylating SGK1, mTORC2 modulates cytoskeletal dynamics, as well as ion transport and growth [60]. Because mTOR is a downstream target of EGFR and MET signaling, it is a promising target for cancer treatment. PI3K pathway abnormal activation is thought to be implicated in a variety of malignancies, and increased PI3K pathway activation is frequently linked to cancer therapy resistance (Figure 1) [61,62].

Ubiquitin modifications control mTOR signaling by degrading protein targets, changing protein cellular location for activation or inactivation of enzyme, stimulating binding of protein to other associated partners, and by additional undiscovered mechanisms. Post translational alterations to the “degron” offer another level of control to the ubiquitination process, this might explain why certain tumors have no inverse correlation/mutually exclusively for E3 ligases and substrates [63]. Surprisingly, ubiquitin-based mTOR regulation appears to be a late evolutionary event for higher eukaryotes as a gained alteration and function, which could help mammals adapt to a more complex and sophisticated biological system. In comparison to the well-studied phosphorylation-mediated control of signaling of mTOR, there is still a lot to learn about ubiquitin’s role in various mTOR signaling components. The discovery of DUBs (deubiquitinases), E3 ligases, and the role of unidentified ubiquitination actions in signaling of mTOR will considerably progress mTOR biology and throw light on medicines that target DUBs and E3 ligases for the treatment of human ailment by modifying activity of mTOR [64,65]. The ubiquitin-proteasome system (UPS) and autophagy are two important intracellular protein degradation mechanisms. Autophagy is characterized by the development of autophagosomes, which merge with lysosomes to generate autolysosomes, which degrade the contents of autophagosomes. The UPS initiates the degradation of short-lived proteins by sequentially adding ubiquitin chains to the target proteins [66,67]. Proteasomes are multicatalytic protease complexes that detect polyubiquitylated proteins [68,69]. Consequently, the UPS and autophagy are linked, and inhibiting one has been demonstrated to have an impact on the other. The evidence for a link between the UPS and autophagy is growing in the literature [70].

## 3. mTOR Inhibitors

mTOR inhibitors can be utilized to treat disorders in addition to the traditional therapeutic indication of organ transplantation. Recent research has suggested that mTOR inhibitors could be used to treat a variety of human malignancies. Rapamycin can, in fact, limit the proliferation of a variety of human and murin cell lines in a concentration dependent manner [71,72]. The therapeutical features of mTOR inhibitors appear to vanish at high drug concentrations, according to a recent experimental investigation, which was validated by a clinical case report, posing a medical difficulty in the treatment of high dosages of rapamycin as well as its derivatives [73]. In light of these assumptions, inhibitors of the pathway of mTOR can be divided into three groups: first, rapalogs, these got their name from rapamycin, second inhibitors with a more heterogeneous mode of inhibition [74]. The second generation of mTOR inhibitors can be classified into two subclasses: (a) mTOR/PI3K dual inhibitors (TPdIs), and (b) mTORC1/mTORC2 dual inhibitors (TORCdIs). This classification is based on the difference in mechanism of action [75]. Rodrik-Outmezguine et al. 2016 linked the 1st and 2nd generation of mTOR inhibitors to create rapalink-1, a third-generation mTOR inhibitor that can aim two targets on the mTOR enzyme at the same time [76,77].

**Figure 2:**
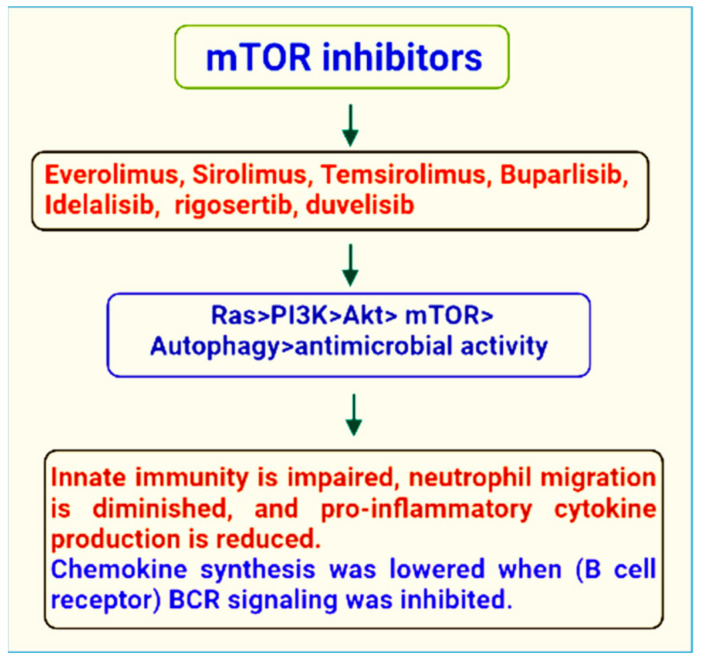
mTOR inhibitors of therapeutic importance.

The first historical inhibitor, rapamycin, inspired the name mTOR. This medication, also known as sirolimus, was first found as a product of the bacteria *Streptomyces hygroscopicus* in a soil sample on the island of Rapa Nui (Easter Island). Rapamycin was first created as an antifungal medication. However, later it was determined that it had significant immunosuppressive and anti-proliferative characteristics [78]. Indeed, mTOR inhibitors are a novel family of medications that are commonly employed in cancers, microbial infections, and immunosuppressive treatments. Their current usage as a viable therapeutic alternative is justified by their good efficacy and low toxicity [79] (Figure 2).

Rapalink-1 is the third generation inhibitor, it lowers the size of the tumor resistant to 1st and 2nd generation inhibitors owing to its significant anticancer action. This strategy offers a novel concept and model for developing new anticancer, antiviral, and antibacterial medications. A variety of medicines have been discovered that suppress mTOR action, in addition to the usual therapeutic mTOR inhibitors [77] (Table 1).

## 4. Therapeutic Role of mTOR Signaling in the Treatment of Microbial Infections

**Figure 3:**
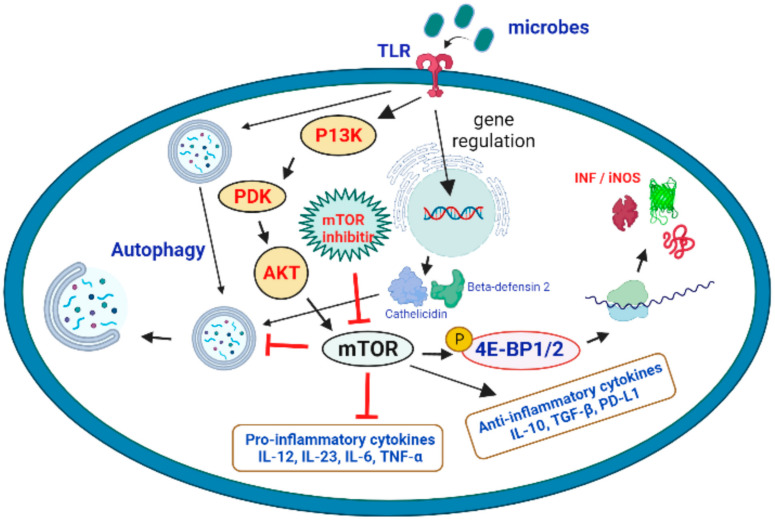
Activation of the mTOR pathway and its potential role in the targeting of microbial infections. Microbial interaction with TLRs activates the P13K > PDK > AKT > mTOR cascade, which results in mTOR activation. It promotes protein translation and the production of iNOS and interferons by phosphorylating 4E-BP1/2 (eukaryotic translation initiation factor 4E binding protein 1 and 2). Microbial detection also promotes autophagy by causing the expression of beta-defensin 2 and cathelicidin. Because mTOR inhibitor is an autophagy inhibitor, it can promote autophagy and fight microbial infections.

mTOR has a crucial function in and against microbial infection, in addition to its eminent role in cell proliferation. The regulation of autophagy, a crucial mechanism in all myeloid and lymphoid cells in all myeloid cells is a key component in the cellular control of mTOR. Pathogen infection-induced stimuli can increase autophagy above basal levels to eliminate intracellular pathogens while also enhancing microbial antigen presentation on cell surfaces to drive the immune response. Infection with the bacteria *Shigella flexneri*, for example, induces amino acid deficiency and consequent mTOR downregulation, resulting in autophagy [22]. The immune system’s ability to recognize and respond to intracellular infections has prompted some viruses to evolve ways to avoid autophagy induction. In order to circumvent immune defense mechanisms, HSV-1 (herpes simplex virus type 1) and HSV-2 (herpes simplex virus type 2) impede autophagy induction [105]. Likewise, *Listeria monocytogenes* and *Salmonella* try to thwart autophagy induction by reactivating mTOR and suppressing the immune response [106]. As a result, these viruses hijack and sustain low levels of autophagy in order to take advantage of the host’s energy and nutrients for their own replication. In such instances, inhibiting mTOR and inducing autophagy above basal levels is advantageous for host defense mechanisms [107]. A cohesive perspective of mTOR-regulated lymphokine expression and surface molecule expression on APCs (antigen-presenting cells) and T cells during infection should assist to better comprehend the immunological and metabolic enigma of mTOR. On a molecular, immunological, and biochemical level, it is yet unknown whether targeting the mTORC1 and mTORC2 complexes with pathogen-derived molecules such as glycoproteins might swing the balance of pro-inflammatory and anti-inflammatory T-cell responses in favor of pathogen survival. Given the mTOR action in T cells and APCs has seemingly different immunological consequences [108], developing medicines that modulate the mTOR signaling axis to combat infectious diseases becomes even more difficult. The principal function of the metalloprotease Gp63 blocking mTOR in APCs in leishmaniosis is to limit translation by activating the translational repressor 4E-BPI. Inhibition of mTOR in T cells, on the other hand, may push them to develop into Treg (regulatory T cells)/Th2 cells (T helper cells), which would likely prolong the infection. As a result, it could be a targeted strategy that combines inhibiting 4E-BP1 in APCs while simultaneously activating mTOR in CD4 T cells to boost the host’s protective Th1 response in the first stages of infection. Because an increase in the quality and quantity of memory T cells is a characteristic of a long-lasting anti-leishmanial immune response, it is possible that pathogen specific recall responses could be improved by targeting mTOR inhibitors to memory cells in vivo in order to speed up their growth. Foxp3 expression can be reduced and tolerance broken by Treg-specific activation of mTOR by blocking upstream inhibitors of mTOR signaling such as TSC1/TSC2 or PTEN. During the later phases of infection, targeting memory cells and Treg cells can be extremely effective. By extension, using the metalloprotease Gp63 alone or in conjunction with other medications might open the door to sensible therapeutic treatments for autoimmune diseases such as multiple sclerosis, where type I interferons exacerbate the condition [109] (Figure 3).

It is worth noting that mTORC1-mediated actions can have both immunosuppressive and immune-activating effects. T-cell energy is increased, Treg expansion is induced, and DC maturation is inhibited by the mTORC1 complex [110]. A recent overview of the role of the mTOR in B-cell growth and function was published [111]. It presents the idea that patients taking mTOR inhibitors may have a hindered innate immune response [112]. mTOR, as well as the generation of proinflammatory cytokines [113,114], is required for neutrophil migration to sites of inflammation [115]. The effect of mTOR inhibition on stromal cells may exacerbate the deficits in innate immunity, resulting in poor wound healing [116]. Stomatitis and pneumonitis occur in a significant number of mTOR inhibitor-treated patients, which may serve as an entrance point for pathogenic bacteria [117]. In a process involving the hypoxia-induced factor 1a (HIF1a) pathway, the mTOR pathway has also been involved in neutrophil activity, including the development of extracellular traps that capture and kill bacteria [118].

It is critical to determine if bacterial pathogens have developed mechanisms that disrupt mTOR-dependent regulation of innate and adaptive immune responses in their hosts. In addition, it would be intriguing to look at how common medications that are used to treat metabolic diseases alter immune responses. For instance, metformin is used to regulate blood sugar levels while simultaneously blocking mTORC1 and possibly inducing inflammation. Given that these drugs impact mTOR activity to improve the host’s innate immune response, it would be intriguing to explore if they can also be used to protect against bacterial infections, or if they can override pathogens’ capacity to modify mTOR activity, such as *Salmonella* [119,120]. Among microorganisms, viruses cannot replicate on their own and must rely on the transcription and translation machinery of the host to duplicate their genome and related proteins. To aid this process, viruses co-opt and modify normal cellular mechanism [121,122]. The regulation of mTOR molecule and its pathways by the severe acute respiratory syndrome coronavirus 2 (SARS-CoV-2) life cycle is of special interest [123]. As mTOR is a serine/threonine kinase that regulates the cellular development, mTOR signaling is also critical for viral translation [124]. In this context, metformin, everolimus, and rapamycin are just a few of the FDA-approved mTOR inhibitors marketed currently. Both DNA viruses such as adenoviruses, cytomegaloviruses, and herpesviruses and RNA viruses such as influenza, HIV, West Nile virus (WNV), and Zika virus (ZIKV) have been shown to affect the mTOR pathway by activating PI3, Akt, or mTOR itself. There are many confirmed evidence that mTOR inhibition reduces viral protein synthesis as well as interferes with virus-mediated transcription activities. WNV, for example, promotes the mTOR pathway via PI3K, prompting downstream protein synthesis activators. WNV-induced mTOR activation was also inhibited by inducible deletion of mTOR cofactors [125].

By modification in the autophagy response, HCV and ZIKV have been presented to increase their own viral reproduction. ZIKV is thought to stimulate autophagy by suppressing the Akt-mTOR pathway, and subsequently hijack the autophagic apparatus to support its own reproduction. In vitro, metformin treatment was reported to suppress ZIKV [125]. HCV can also modify mTOR at several places along the route, uses the autophagic mechanism to generate an infection, which subsequently leads to an increase in hepatocyte formation, allowing for a long-term infection. Metformin decreases the development of infected cells by HCV and inhibits replication of HCV through mTOR pathway, according to multiple studies [126,127]. The influenza A virus has been shown to hijack the Akt/mTOR signaling pathways in order to boost viral replication, with mTOR inhibition greatly restricting viral replication [128]. Buformin (an mTOR inhibitor) has been associated with an improved survival in influenza animal models [14]. In a clinical trial involving 200 patients with H3N2 influenza, treatment with the biguanides phenformin and buformin was found to decrease the incidence of influenza by 5.4% compared to the control group (24%). In randomized controlled studies, mTOR inhibition has also been shown to improve clinical outcomes in H1N1 influenza patients who required mechanical breathing [30,113,129]. mTOR inhibition using guanidine (a metformin derivative) has been associated to poliovirus cytopathic effects in vitro and in primate models [114,130]. Guanidine has also been demonstrated to suppress enterovirus [131,132]. Modulation of the mTOR pathway has been seen in viruses that are more closely linked to SARS-CoV-2. MERS-CoV infection revealed mTOR pathway modification, according to a kinome study. In vitro MERS-CoV investigations have shown that PI3K/Akt/mTOR signaling pathways are significant in viral pathogenesis. Pre-infection suppression of the mTOR pathway resulted in a 60% decrease of MERS-CoV infection in vitro [133]. So, mTOR–PI3K–AKT pathway proved as a critical signaling route in SARS-CoV-2 infection. The scientists tested three mTOR inhibitors in vitro and found that nanomolar medication doses of each agent significantly inhibited SARS-CoV-2 virus [134].

Multiple pathways demonstrated in vitro modification during the time of virus progression in infection, according to a proteo-transcriptomics investigation of infected cells by SARS-CoV-2. All of the linked pathways, in particular, converge on mTOR signaling, although it is unknown how each of them contributes to the viral life cycle or if they help with viral replication and growth [135]. If SARS-CoV-2 cycle and manipulation of cellular pathway is comparable to that of MERS-CoV and other RNA viruses, this could be a promising target for new or repurposed therapeutics. Targeting viral transcription, translation, or both, according to biophysical modelling of SARS-CoV-2, is a great sensitive target for therapeutic suppression and is expected to result in viral replication inhibition. A human protein–protein interaction map for SARS-CoV-2 was created recently in order to identify the possible therapeutic targets. In the cellular response to SARS-CoV-2, the PI3K/Akt/mTOR pathway is crucial. More biochemical research and clinical studies are urgently needed to better understand the specific function of mTOR inhibitors and modulators in the treatment of COVID-19 [55,136,137].

## 5. mTOR in Inflammatory Bowel Diseases

IBD is a complicated immune-mediated inflammatory diseases marked by epithelial barrier failure, dysbiosis, and deregulation of the mucosal immune response [138]. It is made up of two disorders: Crohn’s disease (CD), which causes inflammation throughout the gastrointestinal tract, and ulcerative colitis (UC), which affects only the colon [139]. IBD has no cure, thus pharmacological and surgical therapies to lower the inflammatory burden and induce and sustain disease reduction are the only options. The basis of treatment is anti-inflammatory pharmaceuticals (such as corticosteroids, aminosalicylates), immunosuppressive drugs (such as azathioprine, mercaptopurine, methotrexate), and biologic treatments (anti-tumor necrosis factor drugs, various anti-interleukin drugs) [140]. However, a large minority of patients do not react to these or other innovative targeted biologic therapy in the expanding repertoire. Furthermore, patients frequently develop severe medication effects or secondary loss of response, resulting in a considerable number of patients requiring surgery [141]. As the world is moving closer to the era of personalized medicine, thinking about how to combine our understanding of dysregulated immune metabolism with disease etiology in IBD could offer an exceptional prospect to adapt treatment based on a patient’s metabolic profile [142]. Immune system cells are extremely active, continually detecting and adjusting to changes in their environment. Leukocytes ability to fine-tune their reactions to exterior dangers is controlled by complicated metabolic pathways. The hubs of these pathways are the mammalian target of rapamycin complex 1 and hypoxia inducible factor, which play a role in coordinating cell activation and production of cytokines. As a result, these compounds are appealing therapeutic targets in the treatment of inflammatory diseases [142].

Inflammasomes are multiprotein complexes that have a direct role in the inflammatory process by causing caspase-1, interleukin 1 (IL-1), and interleukin I8 (IL-8) to become overactive [143,144,145,146]. Inflammasome development is aided by five pattern recognition receptor (PRR) candidates. The nucleotide binding oligomerization domain (NOD), absent in melanoma 2 (AIM2), pyrin, leucine-rich repeat (LRR) containing protein (NLR) family (NLRP1, NLRP3), as well as additional PRRs such as NLRP2, NLRP6, NLRP7 and NLRP12, are among these members [147]. Gout [148], hepatitis [149], and ulcerative colitis [148] are all linked to NLRP3 inflammasome assembly and activation [144]. Signaling crosstalk between AMPK, mTOR, and NLRP3 inflammasomes is thought to exist. Both mTOR and NLRP3 activity are reduced when AMPK is activated [150]. As a result, increasing AMPK activity and inhibiting mTOR/NLRP3 signaling could be a promising therapeutic strategy for reducing inflammation.

Several human disorders, comprising IBD affect autophagy. Autophagy dysfunction is linked to alterations in both innate and adaptive bacterial clearance abnormalities in the immune response, goblet/Paneth cell dysfunction, all of which are linked to IBD pathogenesis. TORC1 inhibitors have shown to be beneficial in IBD models. Second-generation mTOR inhibitors that target the kinase domain of the protein have been found to be more effective. Proteasome or histone deacetylase inhibitors coupled with autophagy inhibitors i.e., hydroxychloroquine or activators i.e., everolimus yield optimal anticancer effectiveness [35]. It has been demonstrated that mTORC1 signaling downstream of IFN protects epithelial DNA damage, keeping intestinal epithelial cells stable and preventing cancer growth during colitis, an inflammatory bowel disease that produces ulcers and inflammation in the digestive tract [18]. Intestinal epithelial cell (IEC) homeostasis is disrupted, which shares a role in the development and establishment of a range of disorders, including IBD. According to this research, proliferation of epithelial cell reduces and cellular loss increases during colitis. The mechanism is uncertain, but this study found that the proinflammatory cytokine IFN plays a part in inhibition of epithelial cell growth in intestinal epithelial cells (IECs) via activating mTORC1. In IEC, inhibiting mTORC1 promotes cell growth while increasing DNA damage. In macrophages, inhibiting mTORC1 significantly lower the expression of proinflammatory markers [151,152].

There are two mechanisms that contribute directly to those findings. First, stimulation of the mTORC1 gene reduces DNA damage in IEC by triggering the double strand DNA breaks (DSB) repair mechanism [153]. Second, mTORC1 limits stem cell growth, resulting in less DNA damage [154]. According to studies, the mTORC1 activation may be required for appropriate cell cycle completion and may also work for DNA damage checkpoints. Likewise, because autophagy initiation promotes stem cell division and hyperproliferation [155], mTORC1 suppression [156] may have a role in the preservation of the intestinal stem cell niche. As a result, mTORC1 activation during colitis might be a part of a mechanism that inhibits the rate of proliferation of IECs living in a hostile environment, such as that generated by high levels of ROS [157]. During inflammation, this activity may be especially crucial for reducing the rate of double-strand breaks (DSB) in the nucleus DNA of intestinal epithelial stem cells [18,158]. Thus, mTORC1 protein is significantly activated in the gut mucosa of IBD patients, and inhibiting mTORC1 signaling has therapeutic benefits in animal models of colitis [159,160,161] (Table 2).

To increase the efficiency of treatment, new therapeutic approaches must be discovered [164]. AMPK is a conserved enzyme that regulates cellular metabolism by increasing glucose and fatty acid absorption while also activating the oxidation process to optimize cellular energy utilization [165]. AMPK activation is also thought to help with a variety of cellular issues such as inflammation, insulin resistance, and improper fat deposition [166]. These metabolic anomalies, as well as prone inflammation and apoptosis, are linked to AMPK’s reduced activation and failure to restore normal cellular energy levels [167].

## 6. mTOR and Colorectal Cancer

According to estimates, CRC is the world’s third most common cause of cancer-related mortality. Despite the rising armament of chemotherapeutic and targeted treatments, at the metastatic stage of the cancer, the total survival percentage after five years is less than 10% [168]. As per the global cancer data, there are around 1.1 million newly diagnosed CRC patients and 550 thousand CRC-related deaths, with the death rate from CRC expected to rise by 60% by 2035 [169,170]. Currently, the main therapeutic choices for people with CRC include surgery, radiation, and chemotherapy. Only 70% of CRCs are resectable, with 25 percent of resected patients having chances of recurrent tumors [171]. Only 75% of CRCs are curable, and up to 25% of resected patients will have recurrent tumors. Despite advances in early detection and therapy, only 63% patients with CRC have a 5-year relative survival rate [172]. Furthermore, rising treatment resistance and side effects from chemotherapy and radiotherapy have ruthlessly lowered the quality of life in survivors. To satisfy the therapeutic needs of CRC patients, novel treatment techniques and medications are urgently needed. In the development of sporadic colon cancer, activation of the PI3K/AKT and Ras/Raf (acronym for rapidly accelerated fibrosarcoma)/MEK (MAPK/ERK kinase)/MAPK (mitogen-activated protein kinase) pathways are usually implicated. The PI3K/AKT pathway activates the Ser/Thr protein kinase mTOR [173,174], which is implicated in cell growth, proliferation, and survival. Rapamycin and its chemical equivalents, everolimus and temsirolimus, which target just the mTORC1 complex, have received a lot of interest in the last 15 years as potential anticancer medicines. However, mTOR inhibition in cancer, especially as a monotherapy, has limited advantages, extending lifespan by only a few months in certain trials [36,175], and other studies have shown the occurrence of drug resistance. For example, compared to untreated samples, colon and breast cancers treated with everolimus for four weeks revealed increased levels of activated AKT [176,177]. The discovery that mTORC2 directly phosphorylates the Ser473 residue of the AKT kinase in a variety of diseases has reignited interest in targeting the mTOR pathway, making mTORC2 a novel therapeutic target [178]. Positive regulation of AKT by mTORC2 implies that mTOR acts both upstream and downstream. ATP analogues are the next family of mTOR catalytic inhibitors, and they block both mTORC1 and mTORC2 kinase activity [49,179]. Due to the simultaneous inhibition of rapamycin-insensitive mTORC2 and rapamycin-sensitive mTORC1 [179], these catalytic inhibitors are anticipated to be more powerful in clinical application [180].

**Figure 4:**
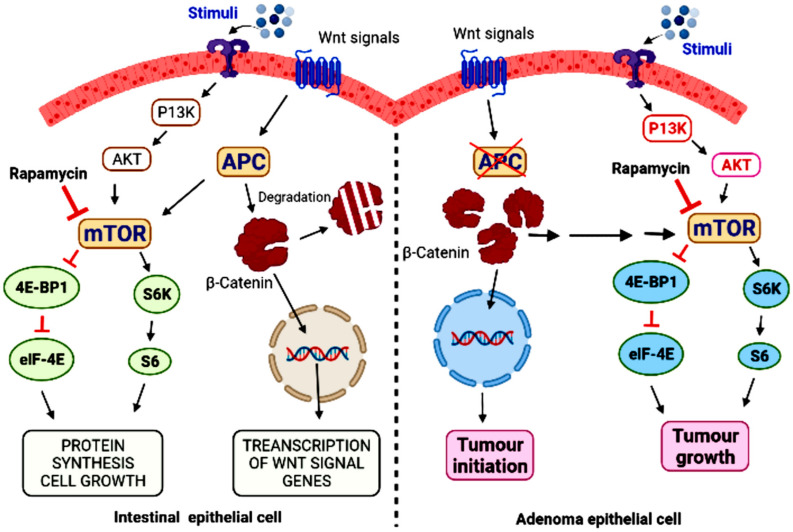
A comparative analysis of adenoma epithelial cell with defective antigen-presenting cells, role of beta-catenin, and rapamycin in the induction and control of cancer respectively. Beta-catenin and rapamycin inhibited cell growth and development of tumor induced by Wnt by inhibiting mTOR signalling.

The use of mTOR inhibitors in CRC treatment might be promising, and the logic for combining them with other chemotherapeutic medications such as irinotecan is reasonable, but it will need the identification of subgroups of individuals who are most likely to respond [177]. Data from both preclinical and clinical research have shown a relationship between mTOR activation and CRC. In APC716 knockout mice (a model for human familial adenomatous polyposis), colonic polyposis was connected to chromosomal instability produced by mTOR activation [180]. In a comparable setting, everolimus inhibition decreased the number and development of polyps [181,182]. The AKT/mTOR pathway is critical for the formation and progression of human CRCs. In a sample of 871 male and 687 female colon cancer patients, it was discovered [183] that genetic diversity linked with the AKT/mTOR pathway contributes to both colon and rectal cancer risk. It has been found that numerous proteins in the mTOR signaling pathway were significantly over expressed in 154 patients of CRC when compared to match normal colonic tissue from the same patients [35,183]. Other studies have demonstrated similar upregulation of mTOR signaling components in human CRCs [184,185,186]. Through the TSC2 complex, a relationship has been demonstrated between Wnt signaling, which is active in most colon tumours, and mTOR [187]. Rapamycin inhibited cell growth and development of tumor induced by Wnt, indicating that it could be used as a treatment [188,189] (Figure 4).

Rapamycin is only effective against mTORC1 [190]. Downregulation of rictor in HT-29 and LS174T, colon cancer cells greatly decreased proliferation of cell, and LS174T cells without rictor proved unsuccessful to progress tumors in a mice xenograft model, implying a function for mTORC2 in CRC [191]. mTORC2 has also been linked to colorectal cancer metastasis [182]. OSI-027 is a mTORC1- and MTORC2-selective inhibitor that has exhibited anticancer properties in a variety of in vivo and in vitro cancer types i.e., human colon cancer xenograft models [192,193], and is at the moment being tested in cancer patients in first phase clinical trials. Although rapamycin analogues (rapalogs) have shown to be effective in preclinical models, their effects have been moderate when utilized as a single-agent therapy [31]. As a result, a new class of anti-mTOR compounds has just been developed. These drugs targeted the mTOR kinase domain, had activity against both mTORC1 and TORC2, and had better anticancer effect compared to rapalogs [194,195,196] (Table 3).

These novel mTOR inhibitors have been shown to be effective in a number of animal models and are currently being tested in a number of clinical trials. In xenograft models, such as colon cancer (LoVo and SW620 cells), AZD8055 (an orally bioavailable, powerful and selective mTOR kinase inhibitor) produced considerable reduction of tumor growth and regression [75].

In a variety of colorectal cancer models, metformin also acts as an adjuvant, apoptosis inducer, antiproliferative, chemopreventive, and radio-chemosensor [213,214]. Metformin mainly affects proteins linked to the cell cycle, via modifying the mTOR-4EBP-eIF4E and MNK1-eIF4GeIF4E axis’ role in signaling. The positive effects of metformin on CRC risk and prognosis may be due to the suppression of MYC protein production [213]. It has been studied in vitro using organoid models of peritoneal metastases from CRC patients, CaCo2 cells, human and mouse LoVo and MCA38 cells, COLO 205 cells, HCT116 cells, Caco-2 cells, DLD-1 cells, SW-480 cells, and RKO cells. It has also been studied in vivo using HT-29-xenografted BALB/c-nude mice, Apc mutated mice, Organoid peritoneal metastases from xenografts of CRC patients, COLO25 and DSS mice, SW48-Mut xenograft nude mice, d HT-29-xenograft SCID mice, PDX-female SCID mice. The use of DMH as a potential treatment in humans was made possible by DMH-induced CRC in diabetic and non-diabetic mice, DMH-induced CRC in rats, and DMH-DSS-induced colitis-associated colon neoplasia in mice [212]. Which eventually led to its application as a potential treatment in patients.

## 7. Conclusions

Among the entire switch of cellular metabolism, mTOR, is a particularly efficient antimicrobial, anti-inflammatory, and anticancer therapeutic target protein. A range of effects can be induced by suppressing the overactive mTOR signaling in these conditions. As a result, numerous mTOR inhibitors are used as medicines in clinical trials. Long-term usage of the identical mTOR inhibitors, on the other hand, will result in the development of tolerance, and as a result, drug resistance; hence, new mTOR inhibitors must be developed. Resistance to anticancer therapies is usually linked to PI3K/AKT/mTOR activation. In preclinical investigations and clinical trials, inhibitors of the PI3K/AKT/mTOR system are being tested to see which types of pathway inhibitors can restore therapeutic sensitivity when used in monotherapy or in a combination. mTOR inhibitors are promising in the treatment of infectious diseases, inflammatory bowel disease, and colorectal cancer.

## Figures and Tables

**Table 1:** mTOR inhibitors and their therapeutic importance.

Sr. No.	Name of mTOR Inhibitor	Therapeutic Importance	Reference
1	Rapamycin (Sirolimus)	Fungicidal characteristics First licensed immunosuppressant for solid organ transplant recipients Antiproliferative properties Anti-aging properties Anti-atherosclerotic properties	[80] [81] [82] [83]
2	Temsirolimus	Treat advanced Renal cancer	[77]
3	Everolimus	Treat Metastatic Renal cell carcinoma (Kidney cancer) Treat Advanced neuroendocrine pancreatic tumours	[84] [85] [86]
4	Ridaforolimus/Deforolimus	Sarcoma Breast cancer	[87] [88]
5	Zotarolimus	Antitumor activity	[89]
6	Torin 1	Suppress colon cancer cells	[90]
7	Torin 2	Antitumor activity	[91]
8	MLN0128	Advanced solid tumors	[92]
9	AZD2014 (Vistusertib)	Metastatic clear cell renal cancer	[92]
10	Voxtalisib (SAR24540; XL765)	non-Hodgkin lymphoma or chronic lymphocytic lymphoma that has relapsed or is refractory	[92]
11	Gedatolisib (PKI-587 PF05212384)	Recurrent endometrial cancer	[92]
12	Rapalink-1	Strong Anticancer properties Tumors resistant to 1st and 2nd generation mTOR inhibitors shrink in size.	[77]
13	Halitulin analog ICSN3250	It has the ability to compete with and interchange phospholipids acid in the mTOR FRB domain	[93]
14	LY3023414	It inhibits the PI3K/mTOR/DNA-PK pathway in tumor cells in vitro It has anti-tumor proliferation effect It causes prolonged reduction in endometrial cancer patients with phosphoinositide-3-kinase regulatory subunit 1 (PIK3R1), phosphate, and PTEN mutations.	[94]
15	O SU-53	Inhibit thyroid cancer cell growth in vitro by activating AMPK to block the mTOR pathway or directly blocking the mTOR pathway.	[95]
16	OSI-027	Anticancer	[96]
17	C C-223	Anticancer	[97]
18	PKI-587	Gastroenteropancreatic Neuroendocrine tumor disease	[98]
19	INK-128	Inhibit angiogenesis and tumor growth in B-cell lymphoma Neuroblastoma Thyroid cancer cells Prostate cancer metastasis B-cell acute lymphoblastic leukemia Breast cancer	[99]
20	GSK2126458	Robust activity in cancer models	[100]
21	XL765	Glioblastoma development is inhibited by triggering ER stress-dependent apoptosis.	[101]
22	NVP-BEZ235	Cancer cell proliferation is inhibited by this compound	[102]
23	P529	Stops cancer cells from multiplying	[103]
24	JR-AB2-011	Anti-glioblastoma multiforme properties	[104]

**Table 2:** mTOR inhibiting drugs for IBD.

Drugs	Mode of Action	References
Mesalamine	Inhibition of mTORC1 signaling pathway	[160]
Tacrolimus,	Inhibition mTORC1 signaling pathway	[161,162,163]
Everolimus	Inhibition mTORC1 signaling pathway	[162,163]
Sirolimus	Inhibition mTORC1 signaling pathway	[142,163]
Methotrexate	Promotes OXPHOS (Oxidative phosphorylation) by activating AMPK and blocking mTORC1	[142]
Corticosteroids	Inhibits glycolysis by blocking mTORC1	[142]
Aminosalicylates	Inhibits glycolysis by blocking mTORC1	[142]
Tacrolimus	Inhibits glycolysis by blocking mTORC1	[142]

**Table 3:** mTOR kinase inhibitors with therapeutic effects in cancer confirmed both in vitro and in vivo.

mTOR Kinase Inhibitors	Drugs	References
Dual inhibitors of mTOR/PtdIns–kinase	NVPBEZ235	[197]
	SF1126	[198,199]
	GSK2126458	[187,200]
	BGT226	[201,202]
	GDC0980	[203,204,205]
mTORC1/mTORC2 dual inhibitors	OSI027	[193,206,207]
	INK128	[208,209]
	AZD2014	[210,211,212]

## Data Availability

All data provided in the manuscript. No additional data available.
